# Surface-Modified Polymeric Nanoparticles for Glioblastoma Therapy: A Review on Targeting Strategies and Delivery of Repurposed Drugs and Off-Label Non-Alkylating Agents

**DOI:** 10.3390/pharmaceutics18040435

**Published:** 2026-03-31

**Authors:** Daniela Maria Sousa, Joana Angélica Loureiro, Maria Carmo Pereira, Maria João Ramalho

**Affiliations:** 1 LEPABE—Laboratory for Process Engineering, Environment, Biotechnology and Energy, ALiCE—Associate Laboratory in Chemical Engineering, Faculty of Engineering, University of Porto, Rua Dr. Roberto Frias, 4200-465 Porto, Portugal; 2 Department of Metallurgical and Materials Engineering, Faculty of Engineering, University of Porto, 4200-465 Porto, Portugal

**Keywords:** nanomedicine, blood-brain barrier, brain tumor, drug repositioning, off-label drugs, drug resistance, cancer therapy

## Abstract

Glioblastoma (GBM) remains the most aggressive primary brain tumor, with poor outcomes under the current standard-of-care with temozolomide (TMZ). Therapeutic failure is multifactorial, mainly driven by TMZ resistance mediated by DNA repair enzymes (MGMT), and an immunosuppressive tumor microenvironment. Drug repurposing and the off-label use of chemotherapeutics have emerged as a strategy to identify non-alkylating agents capable of bypassing MGMT-mediated resistance in GBM. Despite their promise, the effective delivery of these drugs to the brain remains a major challenge due to the low-permeability nature of the blood–brain barrier (BBB). Thus, surface-modified polymeric nanoparticles (NPs) have emerged as adaptable platforms for encapsulating chemically diverse payloads, thereby improving their pharmacokinetics and enabling controlled release at the tumor site. This review critically analyzes ligand-functionalized polymeric NPs for GBM therapy and discusses the integration of repurposed and off-label non-alkylating agents with nanocarrier engineering, focusing on non-alkylating agents as they are MGMT-independent candidates. Furthermore, this review synthesizes recent advances in ligand-functionalized polymeric nanoformulations encapsulating non-alkylating agents for GBM, critically outlining their targeting and transport strategies, design and validation challenges, and future directions. Across the included studies, receptor-targeted surface engineering frequently enhances cellular uptake and in vitro efficacy.

## 1. Introduction

Glioblastoma (GBM) is the most aggressive primary brain tumor, characterized by rapid growth, invasive behavior, and high resistance to conventional treatments [1]. Despite current treatments consisting of surgery, radiotherapy (RT), and chemotherapy (CT) with temozolomide (TMZ), along with advances in other therapies, the median survival for GBM patients remains just over a year [2]. A major challenge in effective GBM treatment is the blood–brain barrier (BBB), which restricts drug penetration into brain tissue, thereby limiting the efficacy of many anticancer agents [3]. Additionally, the low half-life, limited bioavailability of TMZ, and multiple resistance mechanisms also pose significant challenges for successful treatment [4]. One of the most relevant intrinsic resistance mechanisms involves the DNA repair protein of O6-methylguanine methyltransferase (MGMT), which can repair the drug-induced DNA lesions, being therefore a determinant factor for chemoresistance in GBM.

The limited success of current therapeutic approaches underscores the need for innovative strategies capable of overcoming the limitations of GBM therapy. In this context, two complementary strategies have been explored, namely the off-label use of non-alkylating chemotherapeutic agents originally approved for other cancers, and drug repurposing of agents indicated for non-oncological therapeutic applications [5]. These approaches seek to identify new therapeutic indications for already approved drugs, thereby reducing development costs and accelerating clinical translation [5,6]. Several non-alkylating compounds have demonstrated anticancer potential against gliomas, particularly those capable of bypassing MGMT-mediated resistance and modulating multiple intracellular pathways [6]. Nonetheless, many of these drugs still face shortcomings such as low bioavailability, low solubility, short half-life and poor permeability across the BBB. Thus, targeted drug delivery systems have emerged as a promising solution to enhance therapeutic specificity, minimize systemic toxicity, and improve drug accumulation within the tumor microenvironment (TME). By selectively targeting therapeutic agents to GBM cells while sparing healthy brain tissue, these approaches aim to enhance treatment efficacy and improve patient survival outcomes [7,8].

Nanobiotechnology has attracted significant attention over the past decade to achieve such targeted delivery. Nanoparticles (NPs) can encapsulate, protect, and transport therapeutic molecules across the BBB, providing controlled, sustained release directly at the tumor site. In particular, polymeric NPs have been extensively explored due to their high structural versatility, tunable physicochemical properties, and ability to encapsulate chemically diverse drugs. Due to their tunable physicochemical properties and surface functionalization potential, polymeric NPs can be engineered to prolong blood circulation time and reduce premature degradation. Furthermore, surface modification with specific ligands, polymers, or biomolecules enables improved BBB penetration and active targeting of GBM-associated receptors, thereby increasing therapeutic precision [9,10].

This review aims to provide a comprehensive overview of recent advances in the design and application of surface-modified polymeric NPs for targeted GBM therapy, providing a summary of relevant studies published between January 2015 and January 2026. The literature search was conducted across PubMed, ScienceDirect, Google Scholar, and Scopus databases using combinations of the keywords “glioblastoma” or “GBM,” “polymeric nanoparticles”, and “surface-modified” or “functionalized” or “ligand” or “target”. Articles were eligible only if they simultaneously met the following criteria: (i) encapsulation of a non-alkylating off-label chemotherapeutic or repurposed drug, (ii) use of polymeric NPs, (iii) NPs’ surface functionalized with a targeting ligand, and (iv) evaluation of the nanosystem in experimental GBM models. Only studies involving pharmacological agents with prior regulatory approval (e.g., FDA- or EMA-approved drugs) were considered, while studies based on natural compounds were excluded, in order to maintain a focus on clinically translatable drug repurposing strategies. Where multiple studies fulfilled these criteria for the same drug, a single representative article was selected, prioritizing the most recent publication.

## 2. Glioblastoma Treatment and Its Limitations

GBM is the most aggressive form of diffuse glioma originating from astrocytic cells, and it is classified as a grade IV tumor according to the World Health Organization (WHO). The number of new GBM cases is estimated at 250,000 worldwide per year, with around 200,000 deaths worldwide, about 15,000 in Europe, and 9000 in the United States [5,11]. Despite available treatments, GBM remains an incurable disease, with a median survival of just 15 months [12].

The standard treatment for GBM, known as the Stupp Protocol, begins with maximal safe surgical resection, which allows for accurate diagnosis, tumor genotyping, and reducing tumor size, followed by RT, with an administered dose of 60 Gy, combined with 6 cycles of CT with high doses of TMZ, an orally administered alkylating agent that works by methylating DNA [7,13,14].

Conventional TMZ therapy carries significant side effects, including nausea, vomiting, fatigue, and hair loss, alongside more severe hematologic effects such as leukopenia, neutropenia, and thrombocytopenia [15]. TMZ also faces challenges with limited bioavailability, meaning that not all the administered dose reaches the tumor effectively. Furthermore, the half-life of TMZ is notably short at approximately 1.8 h, which results in rapid clearance from the bloodstream, limiting the drug’s availability for prolonged therapeutic effects and requiring high and frequent dosing to maintain efficacy, which increases the side effects [16].

### 2.1. Therapeutic Barriers

#### 2.1.1. BBB—Structure and Selectivity

The BBB is a specialized interface between the systemic circulation and the Central Nervous System (CNS) that maintains brain homeostasis and protects neural tissue from circulating toxins and pathogens [17,18]. It is primarily formed by a monolayer of brain microvascular endothelial cells sealed by tight junctions that restrict paracellular passage of most of the hydrophilic molecules and large lipophilic molecules [17,19]. These endothelial cells are structurally and functionally supported by pericytes, astrocytic end-feet, and perivascular macrophages, which line the basal lamina and perivascular space, contributing to the mechanical rigidity and barrier properties of the vascular wall [17]. This multicellular organization contributes to the structural integrity of the BBB and plays a critical role in its highly selective permeability profile [17].

Free diffusion through this interface is restricted to lipophilic compounds with a molecular weight below approximately 400 Da, while larger molecules required for brain function rely on active carrier-mediated and receptor-mediated transport processes located on the apical surface of endothelial cells [17]. In addition, BBB permeability is strongly influenced by molecular size and hydrophilicity, and most small-molecule drugs, as well as almost all macromolecular therapeutics, do not reach the CNS at effective levels [20].

However, in the context of brain tumors such as GBM, BBB integrity is often disrupted, originating a blood–brain tumor barrier (BBTB). Although in some regions of the BBTB an increased vascular permeability can be observed due to the abnormal endothelial architecture and compromised tight junctions, this disruption is highly heterogeneous. Consequently, drug distribution within the tumor is very heterogeneous, as regions where the BBB is disrupted show higher permeability, while infiltrative tumor margins often retain an intact or partially functional BBB. This heterogeneity limits uniform drug accumulation across the tumor mass and represents a major challenge for effective therapeutic delivery [21].

#### 2.1.2. TMZ Resistance in GBM Patients

Since TMZ received FDA (Food and Drug Administration) approval in 2005, its effectiveness has been compromised in many GBM patients. Around 50% of patients fail to respond to TMZ therapy due to the presence of elevated levels of MGMT, an enzyme that repairs the TMZ-induced DNA damage [5]. MGMT promotes TMZ resistance as it directly removes the methyl group from the O6 position of guanine [22]. This single-use repair mechanism consumes one MGMT molecule per repair event, thereby counteracting TMZ-induced cytotoxicity. High MGMT expression in tumor cells correlates with resistance to TMZ, as the enzyme prevents the accumulation of lethal DNA lesions. Consequently, patients with elevated MGMT levels exhibit poorer clinical outcomes [22,23].

#### 2.1.3. Tumor Microenvironment: Hypoxia, Invasiveness, and Inflammation

Beyond the physical and molecular barriers imposed by the BBB and MGMT-mediated resistance, the GBM TME represents an additional barrier that also limits therapeutic success, which is characterized by low-oxygen regions (hypoxia), changes in the extracellular matrix (ECM), and a chronic inflammatory but strongly immunosuppressive environment. Together, these features increase tumor heterogeneity, promote resistance to therapy, and favor early relapse [24,25].

Hypoxia results from rapid tumor growth and abnormal, poorly organized vasculature, leading to uneven oxygen deprivation. This hypoxic condition stabilizes hypoxia-inducible pathways, pushes tumor cells towards a more glycolytic metabolism, helps to maintain treatment-resistant glioma stem-like cells, and shifts infiltrating immune cells towards an immunosuppressive state [25,26]. Simultaneously, GBM cells actively remodel the ECM to create a mechanically permissive scaffold that facilitates diffuse and infiltrative migration into the surrounding brain tissue and contributes to local recurrence after multimodal therapy [24,27]. The inflammatory compartment of the GBM TME is largely composed of tumor-associated microglia and macrophages with tumor-supporting and immunosuppressive functions. These cells release cytokines and growth factors that promote angiogenesis, ECM degradation, and inhibition of cytotoxic T-cell activity, thereby creating an immunosuppressive TME that responds poorly to current immunotherapies. Collectively, these TME features form a second barrier beyond BBB crossing, restricting NP penetration and intratumoral drug exposure [24,25,26].

## 3. NP-Based Drug Delivery Systems

Nanotechnology-based delivery systems have proven to be a promising strategy to overcome the physiological and molecular barriers that limit conventional therapies, enabling targeted, controlled, and efficient transport of drugs across the BBB and into GBM tissues [28]. In this context, NPs are small-sized carriers, typically 1–1000 nm in size, which can enhance the delivery and stability of therapeutic agents within the brain. Their small size allows for enhanced permeability, enabling them to cross the BBB via receptor-mediated transport, adsorptive-mediated transcytosis, or passive diffusion [8,20].

NPs can encapsulate either hydrophobic or hydrophilic drugs, as well as unstable drugs, protecting them from degradation until they reach their target cells [8]. In cancer treatment, drug resistance is often mediated by P-glycoprotein pumps and limits the effectiveness of many conventional CT agents in GBM [8,18]. However, when encapsulated in NPs, drugs can bypass these efflux mechanisms, leading to a higher concentration of the therapeutic agent within tumor cells [8,18]. This encapsulation thus increases the cytotoxicity of chemotherapeutic drugs on GBM cells, even overcoming resistance to some extent [19]. Furthermore, NPs offer controlled and sustained drug release, reducing the frequency of drug administration and maintaining therapeutic levels in the target tissue for an extended period, minimizing fluctuations in drug concentration, lowering toxicity risks to healthy cells and providing a more consistent therapeutic effect [8,20].

**Figure 1:**
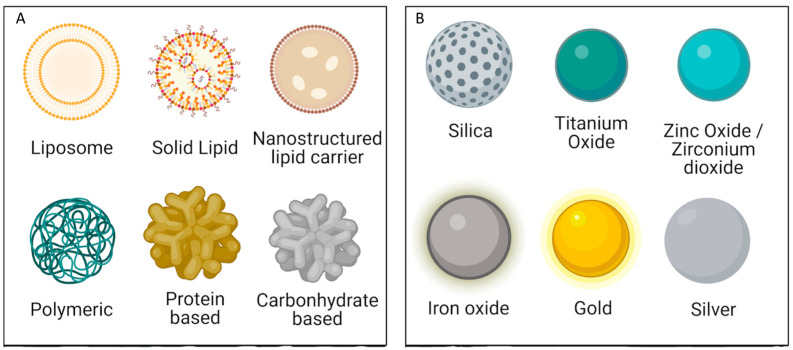
Different types of (**A**) organic and (**B**) inorganic NPs. Adapted from [29].

Although a wide range of NPs are available, as illustrated in Figure 1, the choice of the most suitable system depends mainly on the physicochemical properties of the drug to be encapsulated and the intended therapeutic application.

Among the different types of NPs, polymeric NPs are widely explored for GBM therapy purposes, as they offer high flexibility in composition, structure, and physicochemical properties [30]. Polymeric carriers can be produced from a wide range of natural polymers (e.g., alginate, chitosan, dextran, hyaluronic acid (HA)) as well as widely used synthetic polymers, enabling broad tuning of formulation attributes within the same carrier class [30]. In addition, polymeric platforms, particularly poly(lactic-co-glycolic acid) (PLGA)-based, are widely employed in biomedical delivery due to their biosafety, biocompatibility, and biodegradability, supporting use in drug delivery systems where carrier breakdown and clearance are desirable [31]. Additionally, polymeric delivery systems typically present terminal surface groups, facilitating functionalization strategies aimed at prolonging circulation and improving biodistribution and tumor accumulation [30].

Carrier composition is a key parameter controlling NPs’ physicochemical features, such as size, surface charge and shape, that regulate NPs’ biological fate after administration. For example, surface charge and hydrophobicity significantly influence NPs’ behavior in the bloodstream, particularly in terms of circulation time and immune recognition. While cationic NPs enhance tumor retention and cellular uptake through electrostatic interactions with negatively charged cell membranes, they also promote non-specific interactions with negatively charged serum proteins, protein corona formation, rapid clearance and increased toxicity. In contrast, neutral NPs exhibit prolonged circulation but limited tumor retention and uptake [32].

Furthermore, by incorporating molecules such as polyethylene glycol (PEG) onto their surface, NPs can avoid immune system detection and prolong circulation time, which further improves their chances of reaching brain tissue [18]. This process, known as PEGylation, provides a “stealth” effect by reducing protein adsorption and subsequent recognition by the mononuclear phagocyte system. This, in turn, extends NP circulation time, enhances accumulation in target tissues, and reduces premature clearance [33,34]. This stealth coating improves colloidal stability, diminishes aggregation in biological fluids, and can extend the systemic circulation half-life of PEGylated formulations several-fold compared with their non-PEGylated form, increasing the likelihood that NPs reach otherwise poorly accessible tumor tissues [33,35]. Very dense PEG layers can, however, hinder NP interactions with target cells and impair cellular uptake, creating a trade-off between maximal stealth and efficient delivery to diseased tissues. The magnitude of PEGylation effects depends strongly on its molecular weight and surface density [34,35].

In addition to surface charge and hydrophobicity, NPs’ size is another critical factor determining systemic distribution and tumor accumulation. NPs used in cancer therapy are extremely small, usually between 100 and 200 nm, allowing them to selectively enter tissues and efficiently cross biological barriers [8]. However, NPs smaller than 100 nm are often considered less advantageous due to their increased toxicity and accumulation in certain organs. Studies suggest that smaller NPs are more rapidly absorbed by cells, potentially increasing the risk of cytotoxicity [36]. Moreover, these very small particles tend to distribute broadly throughout the body, accumulating in organs such as the liver, spleen, and lungs, which may lead to adverse effects [28,36].

**Figure 2:**
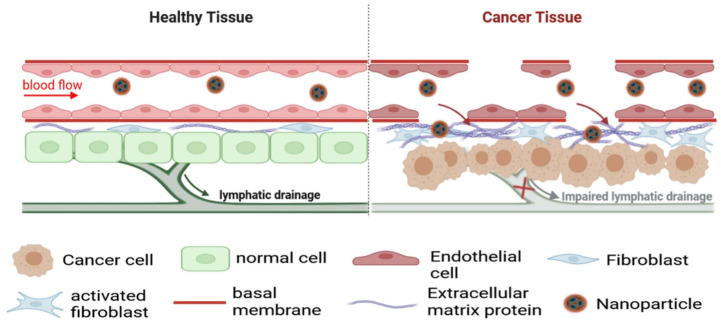
Illustration of the EPR effect. Adapted from [38].

On the other hand, NPs with sizes between 100 and 200 nm have demonstrated more effective tumor retention, achieving passive accumulation in tumor tissues through the enhanced permeability and retention (EPR) effect (Figure 2). This phenomenon occurs due to the irregular and permeable vasculature of tumors, enabling NPs to accumulate more readily in tumor regions compared to healthy tissues. Additionally, NPs in this optimal size range remain in circulation for extended periods, increasing the likelihood of targeting tumors while minimizing accumulation in non-target organs [8,37].

However, passive accumulation through the EPR mechanism alone is insufficient to ensure efficient delivery across the BBB. NP performance can be further improved by surface functionalization that enables active targeting of tumor-specific receptors [39]. Active targeting, by contrast, uses surface modification with ligands, peptides, or antibodies that recognize overexpressed receptors on BBB endothelial cells or GBM cells, enabling receptor-mediated transcytosis and improved tumor specificity [9].

### Surface Functionalization Strategies for BBB Crossing and GBM Targeting

NPs can be functionalized with targeting ligands, such as antibodies or cell-penetrating peptides (CPPs), to improve BBB penetration and tumor cell targeting [8]. The attachment of specific ligands or antibodies to the surface of NPs increases specificity for cancer cells, interacting with receptors that are overexpressed in tumors and promoting cellular uptake through receptor-mediated endocytosis. This selective targeting is critical, as it allows NPs to deliver therapeutic agents specifically to cancer cells, reducing the exposure of healthy brain cells and decreasing systemic side effects [40].

For crossing the BBB, some of the most commonly explored ligands are those that recognize receptors on cells of this barrier, such as insulin and insulin-like growth factor ligands for the insulin receptor or ligands for the LDL receptor [41]. Beyond these, other ligands are being investigated to diversify BBB-targeted nanocarrier systems and develop dual-targeting strategies that exploit receptor overexpression on both brain endothelial and GBM tumor cells. Widely used examples are ligands against the transferrin receptor (TfR), due to the overexpression of this receptor in both the BBB cells and GBM cells [30,41]. In this context, a wide range of ligands has been explored, such as native transferrin (Tf) and monoclonal antibodies against TfR, particularly OX26 and other antibody clones such as RVS10. These have been conjugated to polymeric NPs to improve their uptake by GBM cells and their transport across in vitro BBB models [19,30]. For instance, PLGA NPs functionalized with the OX26 monoclonal antibody against TfR showed increased uptake in human GBM cells and greater transport across an in vitro BBB model than non-functionalized NPs, supporting the importance of TfR-mediated active targeting for drug delivery to GBM [19]. Additionally, short TfR-binding peptides, such as CRT, have been attached to polymeric nanocarriers, increasing NP permeation across BBB models and boosting internalization by GBM cells. These peptides offer the added benefits of lower immunogenicity and less impact on NP size compared to full antibodies [42].

In addition, lactoferrin (Lf) is also suitable for dual targeting of the BBB and GBM cells, as it exploits receptor-mediated transcytosis by interacting with lipoprotein receptor-related protein-1 (LRP1), which is overexpressed on both BBB endothelial cells and glioma cells. Lf-conjugated polycaprolactone NPs were developed and successfully enhanced NPs’ accumulation in GBM cells. Lf-conjugated PLGA NPs successfully crossed the BBB and accumulated in the brain tissue of Sprague Dawley rats [43].

In addition to BBB-directed strategies, several GBM-associated receptors and stem-like cell markers have been exploited to guide NPs selectively into tumor cells, thereby enhancing intratumoral drug accumulation and limiting exposure outside the target [9,30]. Antibodies and peptide ligands recognizing overexpressed receptors such as endothelial growth factor receptor (EGFR), interleukin-13 receptor subunit alpha-2 (IL-13Rα2), and the adhesion molecule CD44 have been conjugated to NP surfaces to promote receptor-mediated internalization specifically into GBM cells [9,44]. Glioma stem-like cell markers, particularly CD133 together with frequently co-expressed partners such as integrin-α6 and CD44, have also been targeted using monoclonal antibody-based constructs and hyaluronic-acid-modified nanocarriers, to concentrate chemotherapeutic agents within resistant, tumor-initiating subsets and thereby reducing recurrence [30,45]. For example, micellar NPs composed of poly(styrene-b-ethylene oxide) (PS-b-PEO) and PLGA were developed and covalently bound to a CD133 aptamer to target the CD133 antigen expressed on the surfaces of GBM stem-like cells. NPs’ conjugation with CD133 aptamer promoted cell killing, providing input for further development for in vivo models [46].

Moreover, integrins such as αvβ3 and α6, which contribute to GBM invasion, vascular remodeling, and stemness, have been addressed by decorating NPs with RGD-containing peptides or other integrin-binding motifs, resulting in reduced glioma cell migration, inhibition of angiogenesis, and improved delivery of cytotoxic or redox-active agents in preclinical glioma models [9,30]. PLA-based nanocarriers were developed and conjugated with RGD for TMZ delivery to GBM tumors. The results revealed improved cellular uptake in vitro and improved efficacy in vivo [47]. Other promising GBM-associated targets, such as MMP-2 and the IL-13 receptor, have also been explored in preclinical NP systems, although they are less extensively investigated than EGFR, CD44, or CD133 [30]. In addition to these targets, folate receptor-directed functionalization using folic acid (FA) is frequently applied to enhance GBM-cell uptake and apparent selectivity, including in ligand-decorated PLGA NPs [31,48,49].

Beyond single-ligand approaches, more advanced nanocarrier systems have been developed, such as dual-targeting systems combining the use of two ligands, one specifically designed to facilitate BBB transport and the other to promote GBM cell recognition. In practice, these nanocarriers incorporate an initial BBB-directed targeting module that promotes NP binding to the brain endothelium and facilitates their passage across the BBB, while a second GBM-directed or microenvironment-responsive elements favor retention and internalization within tumor tissue. In more complex multifunctional designs, dual-targeting modules are further integrated with stimuli-responsive components, such as pH- or redox-sensitive shells, and imaging labels, enabling on-demand drug release and non-invasive monitoring of NP distribution and therapeutic response [50].

All mentioned surface functionalization strategies for NPs are summarized in the table below (Table 1).

**Figure 3:**
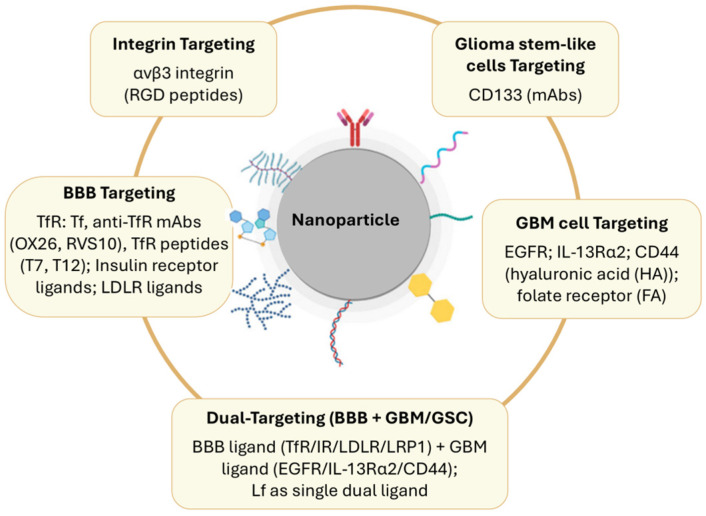
Schematic illustration of the NPs’ surface functionalization strategies for crossing the BBB and for GBM targeting in polymeric NPs.

In addition, Figure 3 provides a schematic illustration of these strategies.

Despite the growing number of targeting strategies and their demonstrated benefits in enhancing BBB transport and GBM specificity, surface functionalization also introduces important translational limitations, including increased formulation complexity, higher manufacturing costs, and potential performance trade-offs. For instance, antibody decoration can slow drug release and consequently reduce in vitro antitumor activity relative to non-targeted formulations. In vivo, ligand-decorated NPs may also lose functional targeting due to protein corona formation, which can mask ligands, change apparent physicochemical properties, and diminish BBB transcytosis efficiency.

## 4. Repurposed and Off-Label Non-Alkylating Drugs Encapsulated in Ligand-Functionalized Polymeric NPs for GBM Targeting

Surface-functionalized success relies largely on the pharmacological agents they deliver. Over the past decade, numerous chemotherapeutics, anti-inflammatory drugs, metabolic modulators, and repositioned agents have been encapsulated into polymeric, lipidic, or hybrid nanocarriers to enhance drug stability, penetration across the BBB, and sustained accumulation within tumor tissue [64].

In the context of GBM, drug repositioning, particularly involving well-established safety profiles, presents a particularly appealing strategy. Many of these agents possess multi-target effects that can modulate tumor metabolism, angiogenesis, or resistance pathways. However, as they were not originally designed for brain delivery, they often present limitations such as poor BBB permeability and suboptimal pharmacokinetics. In parallel, the off-label use of conventional non-alkylating chemotherapeutics has also been explored for GBM treatment. Although these agents may bypass MGMT-related resistance mechanisms, they exhibit high systemic toxicity and limited efficacy [65]. Therefore, the use of NP-based delivery systems emerges as a promising approach to overcome these challenges by improving drug bioavailability, enhancing BBB penetration, and enabling targeted delivery to tumor cells.

Table 2 summarizes published studies that simultaneously employ polymeric NPs functionalized with a targeting ligand and encapsulating non-alkylating agents, with evaluation in GBM models.

### 4.1. Endogenous Ligand or Small-Molecule Targeting

The TfR is highly expressed both at the BBB and in rapidly proliferating tumor cells, making it one of the most widely explored targets to enhance NP transport into the brain and improve tumor cell uptake. Tf, as its natural endogenous ligand, enables receptor-mediated transcytosis across cells expressing this receptor [72]. Tf-conjugated gemcitabine-loaded PLGA NPs were produced and evaluated not only GBM cellular response but also in vivo PK [51]. Gemcitabine is a nucleoside analog used as first-line therapy in pancreatic, lung, and bladder cancers, and has been considered for GBM treatment due to its ability to inhibit DNA synthesis and impair tumor cell replication [73]. The formulation displayed a mean diameter of 143 nm, PDI of 0.21, and zeta potential of −25 mV, with 77.5% encapsulation efficiency and sustained release in PBS over 24 h. In human GBM cells (U87MG), Tf functionalization increased intracellular accumulation and apoptosis (61.3% for Tf-NPs vs. 31.6% for non-targeted NPs). Notably, an in vivo PK study using albino Wistar rats indicated markedly increased brain exposure after oral administration of 10 mg/kg NPs in PBS (e.g., brain Cmax 201.26 μg/mL for Tf-NPs vs. 39.34 μg/mL for free drug and brain AUC0-t 1053.5 vs. 94.37 μg·h/mL). However, the formulation was not evaluated in an in vivo glioma tumor model, neither did the authors evaluate the colloidal stability of the developed NPs under storage or physiological conditions.

The folate receptor is also frequently overexpressed in glioma cells and tumor-associated macrophages and has therefore been extensively explored to promote receptor-mediated NP internalization and improve selectivity [74]. Engineered FA-functionalized PLGA NPs were developed to encapsulate fluoxetine, a selective serotonin reuptake inhibitor primarily used in the treatment of depression and anxiety disorders. Based on its documented effects on cellular signaling and apoptosis-related pathways, it has recently been explored as a repurposing candidate for GBM, with emerging evidence suggesting anti-tumor activity in GBM models [75]. The developed NPs exhibited sizes of 167 ± 8 nm, PDI of 0.23 ± 0.07, and zeta potential of −22.2 ± 0.3 mV with 44.4 ± 3.8% encapsulation efficiency and 3.1 ± 0.3% loading capacity [31]. FA conjugation at the NP surface was confirmed by FTIR, and the formulation exhibited sustained colloidal stability and prolonged release under simulated conditions for 17 days, with FA conjugation reducing early release relative to non-conjugated NPs. Additionally, the nanoformulation proved to be stable in storage conditions for up to 10 weeks. Notably, FA conjugation increased uptake across multiple GBM lines (U251/U87/T98G) and receptor involvement was supported through a folate-blocking/saturation assay. While free fluoxetine displayed lower IC50 values than the encapsulated drug (consistent with controlled release), FA functionalization improved apparent selectivity, with higher IC50 in astrocytes than in GBM lines, and both NP formulations acted as chemosensitizers to TMZ in TMZ-resistant lines. As in many included systems, in vivo efficacy and BBB-transport validation remained unaddressed.

FA functionalization was also applied to repurposed anti-inflammatory drugs with anticancer activity. Prednisolone, a glucocorticoid traditionally used for edema management in glioma patients, has shown therapeutic relevance in other CNS malignancies, supporting its repositioning potential [76]. FA-PLGA NPs loaded with prednisolone were developed, presenting an average size of 326 nm, a zeta potential of −11.9 mV, and a drug loading of 104 µg/mg of NPs [71]. Authors mentioned that NPs were PEGylated (PEG 5000) to improved NP stability and controlled release behavior. However, colloidal stability was not experimentally evaluated under storage or physiological conditions. Quantitative uptake studies in folate receptor-expressing cell lines, including C6 and U87 glioma cells and RAW 264.7 macrophages, showed significantly higher internalization (*p* < 0.01) of FA-functionalized NPs compared to non-conjugated NPs, with increases of approximately six-fold in C6, 2.4-fold in U87, and 5.9-fold in macrophages. Competitive binding assays using excess free FA led to a significant reduction (*p* < 0.05) in NP uptake, supporting a folate receptor-mediated endocytic mechanism. Functionally, FA-NPs showed improved attenuation of pro-inflammatory cytokines compared to the free drug and produced a more sustained inhibitory effect on glioma cell viability. While the cytotoxic effect of free prednisolone on C6 cells decreased after 72 h and was less pronounced in U87 cells, FA-functionalized NPs maintained significantly higher and prolonged inhibitory activity in both models, highlighting their potential as an effective delivery platform.

Some authors have attempted to sequentially address both BBB transport and tumor uptake through multi-receptor targeting. In fact, Kuo and Chen (2015) implemented a dual-ligand (FA and Lf) strategy for etoposide-loaded PLGA NPs to sequentially address BBB passage and tumor-cell uptake [49]. Etoposide is a topoisomerase II inhibitor commonly used in lung cancer and hematological malignancies. It has been investigated as a potential therapeutic option for GBM due to its ability to induce DNA damage and apoptosis in rapidly proliferating tumor cells [77]. Using an in vitro human BBB model (co-culture of HBMECs and astrocytes), the dual-ligand formulation approximately doubled the etoposide permeability coefficient versus non-targeted PLGA NPs while maintaining the barrier integrity. The system achieved sub-200 nm sizes (~83–182 nm) with high encapsulation efficiency (approaching ~100% under optimized surfactant conditions) and sustained release over ~31 days, and it improved antiproliferative efficacy in U87MG relative to controls. The authors suggest that the zeta potential values higher than 30 mV may indicate adequate colloidal stability, this was not experimentally validated under storage or physiological conditions. Also, the study did not evaluate efficacy in an in vivo GBM model or assess clinically relevant dosing and PK.

### 4.2. Antibody-Mediated Targeting

Beyond endogenous or small-molecule ligands, several studies explored the use of antibodies to address tumor heterogeneity and sequential delivery barriers. Antibody-mediated targeting offers high specificity by recognizing tumor-associated antigens, enabling selective NP binding and internalization in heterogeneous GBM cell populations. Dual-antibody targeted PLGA NPs were developed, conjugating panitumumab, an anti-EGFR antibody, plus an antibody against the programmed death-ligand 1 (PD-L1) [68]. The dual-antibody approach was intended to exploit the frequent co-expression of EGFR and PD-L1 in aggressive GBM subpopulations [78]. The NPs were further loaded with docetaxel, a second-generation taxane approved for the treatment of breast, prostate, and lung cancers, which has been explored for GBM due to its potent disruption of microtubule dynamics and associated anti-proliferative activity [79]. The developed NPs showed a mean size of 124 ± 1 nm NPs, a PDI of 0.18 ± 0.02 and a zeta potential of −24.6 ± 3.1 mV, with the authors reporting a conjugation efficiency up to 76.2 ± 6.2%. Dual targeting increased endocytosis (9.2-fold uptake increase in U87-MG vs. non-targeted NPs) and improved cytotoxicity and apoptosis compared with single-ligand systems and mixtures. However, validation remained in vitro, with no in vivo GBM/BBB evaluation. Additionally, the formulation demonstrated short-term stability under storage conditions in a freeze-dried state, which is advantageous for potential therapeutic applications. However, this evaluation was limited to 24 h and did not include assessment under physiological conditions. Furthermore, the incorporation of dual antibody ligands increases formulation complexity, which may pose challenges for large-scale production and affect batch-to-batch consistency.

Other authors also pursued an antibody-driven strategy to produce NPs for the delivery of letrozole [69], an aromatase inhibitor widely used in hormone receptor-positive breast cancer. Its ability to modulate estrogen-related signaling pathways has led to its investigation as a therapeutic candidate for GBM [80]. The authors proposed PLGA NPs conjugated with the antibody ch14.18/CHO against a tumor-associated antigen, the disialoganglioside GD2, to exploit its high expression in GBM cells. The developed NPs showed mean sizes of 144 ± 28 nm and a zeta potential of −21.6 ± 0.6 mV. Additionally, the NPs exhibited high encapsulation efficiencies (82.2 ± 5.8%) and a suitable release profile reaching ~50% of drug release over ~50–72 h. In a GBM-colorectal co-culture, anti-GD2 functionalization yielded selective localization in GBM cells but not in HT29 cells, supporting GD2-mediated specificity, and the authors further linked aromatase inhibition to a miR-191-associated mechanism in patient-derived GBM models. A notable translational constraint was the reported size increase after antibody conjugation (>360 nm discussed), which may compromise BBB penetration and reinforces the need for optimization of in vivo brain tumor delivery. Additionally, the colloidal stability of the nanosystem under physiological conditions or the potential formation of a protein corona were not addressed, despite the fact that these can alter NP surface properties and affect targeting efficiency.

### 4.3. Advanced Ligand-Based Strategies

Beyond classical single-receptor targeting approaches, several studies have explored more complex surface-engineering strategies to address multiple biological barriers simultaneously or to incorporate active therapeutic ligands.

A representative example is the use of RGD peptide functionalization to target integrin αvβ3, combined with an intranasal nose-to-brain delivery strategy to overcome BBB limitations. Integrin αvβ3 is frequently overexpressed in GBM cells and tumor-associated vasculature, where it plays a central role in tumor angiogenesis, invasion, and cell adhesion, making it an attractive target for receptor-mediated NP delivery [81]. RGD-functionalized PLGA NPs were developed to exploit integrin αvβ3 binding and overcome BBB limitations through intranasal administration [66]. The authors proposed this nanosytem for the delivery of doxorubicin, an anthracycline indicated for several solid and hematological malignancies, which has most recently gained interest for application in GBM [82]. However, it is limited by poor brain penetration, creating the need for alternative delivery strategies. The developed NPs were ~180–200 nm (PDI of ~0.1) with an initial burst release (~25% in the first 4 h) followed by sustained release. RGD functionalization increased intracellular delivery and apoptosis in C6 glioma cells. Furthermore, in vivo studies using male Sprague–Dawley rats with orthotopic C6 xenografts revealed that intranasal administration of nanoformulations containing 0.35 mg/kg of equivalent DOX reduced tumor growth and tissue staining results consistent with efficacy, showing increased tumor-cell apoptosis and reduced tumor-cell proliferation. Among the studies reviewed, this work uniquely combines RGD-mediated active targeting with intranasal nose-to-brain delivery and demonstrates tumor growth inhibition in an intracranial C6 GBM rat model. The colloidal stability of the developed NPs was not evaluated under storage or physiological conditions, which may limit the prediction of their performance in clinically relevant settings.

While integrin-targeted systems aim to enhance tumor-cell binding and improve brain delivery through alternative administration routes, other approaches have focused on engineering surface properties to mimic endogenous transport mechanisms and enable microenvironment-triggered activation. A polymeric, dual-responsive paclitaxel system intended to improve BBB transport, tumor activation, and penetration was developed [67]. Paclitaxel is a microtubule-stabilizing agent extensively used in breast, ovarian, and lung cancers, and has attracted attention as a candidate for GBM based on its strong anti-mitotic effects and capacity to suppress tumor cell proliferation [83]. The developed nanoplatform comprised a chitosan-paclitaxel prodrug core (disulfide linkage) self-assembly coated with a pH-labile shell composed of poly(2-methylacryloxyethyl phosphocholine) (Schiff-base), that serves as a choline-analog “ligand-mimetic” intended to support BBB transport. NPs were ~24 nm in size and shell-coated NPs ~68 nm, with charge shifting from ~ +30 mV to ~ +8 mV after coating. The nanosystem demonstrated good colloidal stability, remaining stable in PBS for five days. Under mildly acidic conditions, shell detachment induced size shrinkage and charge reversal, consistent with microenvironment-triggered activation. Cytotoxicity followed this switch (high IC50 at pH 7.4 vs. markedly lower IC50 at pH 6.5), and BBB involvement was supported through a transwell model where permeability decreased upon choline uptake inhibition (HC-3). Animal studies were conducted in female nude mice bearing orthotopic U87MG xenografts. The developed formulations were administered intravenously every three days for a total of seven doses, at a paclitaxel-equivalent dose of 5 mg/kg. The obtained results revealed that the developed nanosystem achieved strong tumor suppression and reported survival benefit in the animals with supportive histology and biosafety readouts. However, the self-assembly-based preparation may present challenges during scale-up, as small variations in processing conditions can affect NP characteristics and batch-to-batch consistency.

In contrast to ligand-mimetic or stimuli-responsive systems, some platforms have incorporated bioactive therapeutic proteins at the NPs’ surface, thereby combining targeting and direct receptor-mediated cytotoxic signaling within a single formulation. In fact, polymeric NPs based on self-assembled amphiphilic poly(N-vinylpyrrolidone were developed and bortezomib was encapsulated and the NP surface was decorated with DR5-B [70]. Bortezomib is a proteasome inhibitor approved for the treatment of multiple myeloma and mantle cell lymphoma. Given its capacity to disrupt protein homeostasis and induce tumor cell apoptosis, it has been explored as a candidate for GBM [84]. DR5-B is a DR5-selective variant of the TRAIL ligand that binds death receptor 5 and actively triggers extrinsic apoptotic signaling, thereby functioning not only as a targeting moiety but also as a bioactive therapeutic protein [85]. DR5-B conjugation increased the hydrodynamic diameter to the submicron range (size of ~690 ± 20 nm) and improved apparent BBB passage in a transwell model, alongside enhanced cytotoxicity in 2D and 3D GBM models. Beyond in vitro testing, an in vivo zebrafish xenograft was also used to support anti-tumor activity. This system is informative because the surface ligand is not only a targeting element but also an active therapeutic protein (DR5 receptor agonist), meaning that the observed effects reflect a combined drug-protein strategy rather than a conventional drug plus inert targeting ligand approach. However, the marked size increase after protein decoration should be considered as a potential constraint for brain-delivery translation. Additionally, the colloidal stability of the nanosystem was not assessed under storage or physiological conditions, which may limit the prediction of its in vivo performance in humans. Moreover, self-assembly approaches introduce additional challenges for large-scale production, as minor variations in formulation parameters can lead to inconsistencies in NP properties.

These more advanced strategies show a progression in the field from conventional single-receptor targeting to multifunctional surface-engineering approaches aimed at addressing the biological complexity of GBM and its delivery barriers.

## 5. Discussion and Concluding Remarks

The studies here reviewed show that ligand-mediated surface engineering of polymeric NPs is used to address two dominant obstacles in GBM delivery, namely the BBB permeation and tumor-selective uptake. The described NPs accommodate chemically diverse non-alkylating payloads, including anticancer drugs (doxorubicin, etoposide, docetaxel, paclitaxel, gemcitabine) and repurposed non-oncological drugs (fluoxetine, letrozole, prednisolone). Taken together, these reviewed studies support the premise that ligand-functionalized polymeric NPs can improve GBM-targeted delivery primarily by increasing receptor-mediated cellular association and uptake, while enabling controlled release for non-alkylating payloads. Within the reviewed works, PLGA is the most explored nanomaterial, reflecting its broad use in drug delivery, biodegradability, and formulation versatility, as well as the practical ease of introducing surface ligands via established coupling chemistries [31,48].

Among the reviewed targeting strategies, FA functionalization appears most frequently, likely because FA provides low immunogenicity, chemical stability, and rapid tumor penetration due to its low molecular weight, while also enabling easy production and quality control [86]. Additionally, it is noted that folate receptor targeting is consistently used to increase uptake in GBM cell models and, when implemented with competition/blocking experiments, provides relatively strong mechanistic support for receptor involvement. However, it is critical that folate receptor expression can vary across glioma contexts, suggesting that folate receptor-targeting may deliver variable benefit across tumors and should ideally be paired with receptor-expression verification in the chosen models and, ultimately, in vivo [87].

Furthermore, the reviewed works demonstrated that antibody-based systems can improve selectivity in heterogeneous tumors such as GBM, particularly when dual targeting is used, but may introduce size and heterogeneity limitations that complicate BBB delivery, reinforcing the importance of pairing ligand choice with the delivery strategy [30,51,66,68,69]. Nevertheless, antibody-based surface functionalization can substantially increase manufacturing and quality-control costs, motivating interest in more cost-effective targeting ligands [30]. Additionally, and in contrast, protein ligands (e.g., Tf and Lf) and especially peptides are typically smaller than antibodies and can support deeper tissue penetration and more scalable synthesis, although they may be less receptor-specific than a monoclonal antibody [88,89,90].

Additionally, several comparatively under-explored directions could plausibly strengthen the next generation of polymeric GBM nanomedicines for drug repurposing. In particular, nucleic-acid aptamers and brain-shuttle peptides (e.g., Angiopep-2, exploiting LRP1-mediated receptor-mediated transcytosis) constitute non-antibody targeting options that can offer smaller ligand formats and BBB-trespassing mechanisms, while aptamers may additionally provide antibody-like affinity/specificity together with advantages such as smaller size and simpler modification. Within the polymeric GBM NP systems review here, these ligand classes appear comparatively less represented than small-molecule ligands (e.g., FA), supporting their prioritization for broader exploration [30,91].

In addition, across the available literature, the spectrum of non-alkylating agents used for polymeric nanoencapsulation in GBM remains incomplete. Therefore, future research should consider additional candidates. For instance, although metformin has not yet been widely investigated in polymeric NP platforms for GBM therapy, a recent non-polymeric nanocarrier study demonstrated that metformin-based nanoformulation significantly reduced viability in U251 and U87 GBM cells, supporting metformin as a plausible repurposed non-alkylating candidate for future evaluation in polymeric NPs for GBM [92]. Furthermore, the highly hydrophilic nature and low molecular weight of this drug can limit its incorporation into hydrophobic polymeric matrices. In fact, using PLGA NPs for other applications (type 2 diabetes) has shown that encapsulation efficiency can vary greatly depending on formulation parameters, with reported values ranging from 15% to 60% when optimized through experimental design [93]. The authors verified that the hydrophilic nature of metformin promotes its diffusion into the external phase during NP preparation, particularly when higher internal aqueous phase volumes are used or when strong homogenization conditions are applied, which increase drug leakage. These findings highlight that production parameters play a critical role in determining loading and release performance for hydrophilic drugs in polymeric NPs.

Similarly, simvastatin and albendazole are non-alkylating candidates with demonstrated anti-glioma activity in vivo when formulated in non-polymeric biomimetic nanocarriers. These include Lf-conjugated NPs co-delivering simvastatin and fenretinide, which showed efficacy in subcutaneous and orthotopic glioma models [94], and menthol-modified albumin/silver NPs delivering albendazole with BBB penetration and enhanced anti-glioma effects [95]. Together, these works support the idea of their testing as repurposed drugs in polymeric NP platforms, such as PLGA, for GBM therapy. Additionally, valproic acid, a non-alkylating class I/IIa histone deacetylase (HDAC) inhibitor, remains comparatively under-explored as a payload in polymeric GBM nanocarriers. This is despite its prior investigation in other glioma-related applications, including a folate-targeted, pH-responsive mesoporous silica valproic acid formulation [96], which increased radiation-induced apoptosis and reduced clonogenic survival in C6 and U87 glioma cells. Although encapsulation of valproic acid into polymeric NPs has not yet been explored for GBM application, its moderate lipophilicity may favor its incorporation into hydrophobic polymeric matrices. However, its relatively low molecular weight can still compromise its retention within the NPs. This may result in faster diffusion through the polymer matrix and relatively rapid release kinetics, potentially leading to burst release and limiting sustained delivery. Interestingly, most reported polymeric NP formulations of valproic acid are based on chitosan systems (although not for GBM application), maybe due to the possibility of electrostatic interactions between the ionized carboxyl group of valproic acid and the cationic polymer, which can improve drug retention [97,98,99].

Lastly, tamoxifen could also be considered for further exploration as a non-alkylating candidate for GBM therapy. Although classically an estrogen receptor modulator, it can inhibit protein kinase C (PKC), frequently overexpressed or hyperactivated in aggressive CNS tumors, including GBM. Thus, by binding to this protein, which regulates cellular processes and influences cell growth and transformation, tamoxifen reduces its activity, downregulating pathways that promote cell cycle progression [100,101]. Most published studies involving tamoxifen-loaded nanoformulations focus on breast cancer applications and, to date, the few reported systems targeting GBM have employed tamoxifen primarily as a surface ligand rather than as the encapsulated therapeutic agent [102,103,104,105]. Nevertheless, tamoxifen remains a promising candidate whose non-alkylating nature warrants further investigation as an encapsulated payload in polymeric NP platforms for GBM therapy.

Furthermore, although most of the nanoplatforms here reviewed demonstrate enhanced receptor-mediated cellular uptake in vitro, recurring limitations across the overall dataset are verified. These include the frequent absence of efficacy studies in orthotopic GBM models, the scarcity of quantitative brain/tumor PK evaluation and the limited in vivo receptor-dependence validation. In addition to biological performance, pharmaceutical aspects such as reproducibility and colloidal stability should also be considered. In more complex systems, such as the ones based on self-assembly or involving multiple functional components, changes in preparation conditions can significantly affect NPs’ properties. Thus, maintaining colloidal stability under physiological conditions remains challenging, as protein adsorption and aggregation may alter NPs’ behavior and reduce targeting efficiency. The majority of the works reviewed here did not evaluate the colloidal stability of the developed NPs in storage or physiological conditions. However, these factors must be carefully considered for a successful clinical translation.

In addition, although polymeric NPs are generally considered biocompatible and biodegradable, surface functionalization can alter their degradation profile and in vivo fate. For example, in the case of PLGA, despite the fact that this polymer degrades into lactic and glycolic acid and is metabolized through natural pathways, factors such as polymer composition, NP size, and surface modifications can influence degradation kinetics and clearance profiles. PLGA NPs with a mean size of 100 nm exhibited prolonged circulation, reduced uptake by the reticuloendothelial system, and increased brain accumulation compared to larger particles (160 nm), demonstrating that appropriate size optimization can enable long-circulating behavior even in the absence of additional surface modification [106].

Furthermore, the presence of surface ligands, especially larger or biologically active molecules such as proteins or antibodies, may affect NP stability, biodistribution, and clearance, potentially leading to prolonged circulation or tissue accumulation [107]. Moreover, surface modification with targeting ligands can promote serum protein adsorption after intravenous administration, leading to the formation of a protein corona that alters NP properties, impacts their biological performance, and poses a major obstacle to targeted delivery. Interestingly, despite the recognized impact of protein corona on NPs’ biological behavior, a recent systematic review, based on 470 studies and 1702 NP systems, showed that only about 20% of the analyzed systems were polymeric NPs, while most focused on metallic nanocarriers [108]. This highlights a major gap in the current research field, especially because careful characterization of NPs after corona formation is essential to minimize their effects and to better understand their impact on targeting performance and long-term toxicity.

The long-term in vivo fate of targeted NPs in brain tissue is still not fully understood, particularly regarding tissue accumulation, degradation behavior, and immune responses. Importantly, surface conjugation with ligands can alter biological interactions, as their nature, size, and density can influence immunogenicity, opsonization, and clearance mechanisms. In particular, protein- or antibody-based ligands may trigger immune recognition or affect NPs’ retention in blood circulation and tissues. For example, the development of antibodies (e.g., human anti-mouse antibodies) can neutralize the therapeutic effect, accelerate systemic clearance, and reduce accumulation at the target site, while also potentially leading to adverse reactions. Strategies such as the use of chimeric, humanized, or fully human antibodies have been developed to avoid immunogenicity, although immune responses may still occur. Additionally, the choice between whole antibodies and antibody fragments is also relevant, as fragments (e.g., Fab, scFv) generally exhibit lower immunogenicity and improved tissue penetration, whereas full antibodies may increase nonspecific interactions and clearance due to Fc-mediated recognition [109]. Thus, surface modification may introduce additional safety concerns, including potential immunogenicity, off-target interactions, and altered clearance pathways, that must be considered to support clinical translation.

To conclude, future work should prioritize orthotopic GBM efficacy studies supported by quantitative brain-tumor PK assessment and in vivo confirmation of receptor dependence (e.g., blocking/competitive inhibition experiments). In addition, greater attention should be given to pharmaceutical aspects, including the reproducibility of NP preparation and the maintenance of colloidal stability under physiological conditions, particularly in complex multifunctional systems. In parallel, expanding the diversity of targeting-ligand classes (including antibodies/antibody-derived formats and non-antibody ligands such as peptides, brain shuttles, and aptamers), together with the continued exploration of non-alkylating payload candidates, will be essential to advance polymeric NP platforms toward clinically translatable GBM therapies. Furthermore, a better understanding of the impact of surface functionalization strategies on long-term safety, immunogenicity, and in vivo fate will be critical to support their successful clinical translation.

## Figures and Tables

**Table 1:** NPs’ surface functionalization strategies for crossing the BBB and GBM targeting in polymeric NPs.

Surface Functionalization Strategy	Example Ligands/Targets	Main Goal	Type of Polymeric NP	Refs.
BBB-targeting (receptor-mediated transcytosis across the BBB)	TfR: Tf, anti-TfR mAbs (OX26, RVS10), TfR peptides (T7, T12); Insulin receptor ligands; LDLR ligands	BBB penetration/transcytosis—higher brain delivery	PLGA NPs; poly(β-L-malic acid) (PMLA) nanoconjugates; dendrigraft poly-L-lysine (DGL) NPs; PEG-PLA micelles; human serum albumin (HSA) NPs	[19,51,52,53,54,55,56]
GBM cell targeting (tumor receptors)	EGFR; IL-13Rα2; CD44 (hyaluronic acid (HA)); folate receptor (FA)	Tumor-cell uptake and intratumoral accumulation—reduced off-target exposure	PLGA NPs; PEG-PLGA NPs; methoxy poly(ethylene glycol)-poly(D,L-lactide) (MPEG-PDLLA) NPs	[31,57,58,59]
Glioma stem-like cells targeting (stem-like subsets)	CD133 (mAbs)	Drug enrichment in resistant glioma stem-like cells—lower recurrence potential	PLGA-PEG NPs	[60]
Integrin targeting (invasion/angiogenesis/stemness)	αvβ3 integrin (RGD peptides)	Anti-migration/anti-angiogenesis effects + improved delivery to invasive niches	PEG-poly(ε-caprolactone) (PCL) NPs	[61]
Dual-targeting (BBB + GBM/GSC)	BBB ligand (TfR/IR/LDLR/LRP1) + GBM ligand (EGFR/IL-13Rα2/CD44); Lf as single dual ligand	Sequential delivery: BBB crossing + tumor/glioma stem-like cells retention and uptake	Chitosan hydrochloride + HA, polysaccharide NPs; linear polyethylenimine (LPEI)-PEG polyplexes	[62,63]

**Table 2:** Summary of the included studies investigating ligand-functionalized polymeric NPs encapsulating non-alkylating agents for GBM targeting. Studies are listed by drug in the order presented and report the NP platform, surface ligand, GBM model used, and overall validation level (in vitro, in vitro BBB, or in vivo).

Drug and Its Classification	Polymeric NP Platform	Surface Ligand	GBM Model	Main Conclusions	Ref.
**Doxorubicin** (Conventional chemotherapy, off-label for GBM)	PLGA NPs	RGD	in vitro (C6 cells) and in vivo (orthotopic C6 rat GBM, intranasal administration)	RGD-NPs enhanced cell apoptosis and reduced tumor growth in vivo	[66]
**Etoposide** (Conventional chemotherapy, off-label for GBM)	PLGA NPs	Lf and FA	in vitro (U87MG; HBMECs and astrocytes for BBB)	Dual-ligand NPs increased in vitro permeability across the BBB model and improved antiproliferative activity in GBM cells	[49]
**Paclitaxel** (Conventional chemotherapy, off-label for GBM)	Chitosan-paclitaxel prodrug core with poly(2-methylacryloxyethyl phosphocholine) (pMPC-CHO) shell	pMPC-CHO (ligand-mimetic)	in vitro (U87MG; bEnd.3for BBB) and in vivo (orthotopic U87MG mouse GBM, intravenous administration)	Dual-responsive NPs enabled microenvironment-triggered activation, improved BBB transport in vitro, and produced strong tumor suppression and survival benefit in vivo	[67]
**Docetaxel** (off-label for GBM)	PLGA NPs	Anti-EGFR (Pmab) and anti-PD-L1	in vitro (U87-MG; A549)	Dual-antibody NPs increased uptake and cytotoxicity compared with non-targeted and single-ligand systems	[68]
**Fluoxetine** (Repurposed, Antidepressant drug)	PLGA NPs	FA	in vitro (U251; U87MG; T98G; NHA)	FA-NPs improved uptake and apparent selectivity toward GBM cells, while acting as chemosensitizers to TMZ in resistant GBM cells	[31]
**Gemcitabine** (Conventional chemotherapy, off-label for GBM)	PLGA NPs	Tf	in vitro (U87MG) and in vivo (rat pharmacokinetics brain/plasma, oral administration)	Tf-NPs increased uptake and apoptosis in U87MG cells and significantly enhanced brain accumulation in vivo	[51]
**Letrozole** (Conventional chemotherapy, off-label for GBM)	PLGA NPs	Anti-GD2 antibody	in vitro (patient-derived GBM lines; GBM–HT29 co-culture)	Anti-GD2-NPs enabled selective localization in GBM cells and linked aromatase inhibition with a miR-191-associated mechanism, although antibody conjugation increased NP size	[69]
**Bortezomib** (Conventional chemotherapy, off-label for GBM)	Poly(N-vinylpyrrolidone NPs	DR5-B (TRAIL variant)	in vitro (U87MG; T98G; in vitro BBB) and in vivo zebrafish xenograft	DR5-B- NPs combined receptor-mediated targeting with direct apoptotic signaling, enhancing cytotoxicity despite an increase in NPs’ size	[70]
**Prednisolone** (Repurpose, anti-inflammatory drug)	PLGA NPs	FA	in vitro (C6; U8MG; RAW 264.7)	FA-NPs enhanced uptake in glioma cells and macrophages and produced a more sustained inhibition of glioma cell viability compared with the free drug	[71]

## Data Availability

No new data were created or analyzed in this study.

## Abbreviations

The following abbreviations are used in this manuscript:

BBB	Blood–brain barrier
BBTB	Blood-brain tumor barrier
CNS	Central Nervous System
CPPs	Cell-penetrating peptides
CT	Chemotherapy
ECM	Extracellular matrix
EGFR	Endothelial growth factor receptor
EPR	Enhanced Permeability and Retention
FA	Folic acid
FDA	Food and Drug Administration
GBM	Glioblastoma
HA	Hyaluronic acid
HDAC	Histone deacetylase
IL-13Rα2	Interleukin-13 receptor subunit alpha-2
Lf	Lactoferrin
LRP1	Lipoprotein receptor-related protein-1
MGMT	O6-methylguanine methyltransferase
NP(s)	Nanoparticle(s)
PEG	Polyethylene glycol
PKC	Protein kinase C
PLGA	Poly(lactic-co-glycolic acid)
RT	Radiotherapy
scFv	Single-chain variable fragments
Tf	Transferrin
TfR	Transferrin receptor
TME	Tumor microenvironment
TMZ	Temozolomide
WHO	World Health Organization
